# Cerebral Embolic Protection: Is There a Benefit for Left Atrial and Mitral Valve Procedures?

**DOI:** 10.1007/s11886-024-02132-4

**Published:** 2024-10-07

**Authors:** Besir Besir, Samir R. Kapadia

**Affiliations:** Department of Cardiovascular Medicine, Heart, Vascular and Thoracic Institute, Cleveland Clinic, 9500 Euclid Avenue, J2-3, Cleveland, OH 44195 USA

**Keywords:** Cerebral embolic protection, Stroke, Mitral transcatheter edge-to-edge Repair (M-TEER), Transcatheter mitral valve replacement (TMVR), Left atrial appendage occlusion

## Abstract

**Purpose of Review:**

This review aims to highlight the current evidence on the use of cerebral embolic protection devices (CEPD) in left atrial and transcatheter mitral valve procedures. It also aims to summarize the antithrombotic management of patients undergoing such procedures.

**Recent Findings:**

Ischemic stroke is one of the most devastating complications of structural heart procedures. The manifestation of periprocedural stroke can range from asymptomatic and detectable only through brain imaging to major stroke with neurological deficits. CEP devices were initially developed to mitigate the risk of stroke associated with transcatheter aortic valve replacement (TAVR). However, the efficacy of such devices during different cardiac interventions is yet to be fully demonstrated, especially in left atrial appendage closure (LAAO), and mitral valve interventions. Few studies demonstrated that the risk of periprocedural strokes after LAAO and mitral valve interventions is not negligible and is highest during the periprocedural period and then falls. The majority of patients undergoing those procedures have cerebral ischemic injuries detected on diffusion-weighted magnetic resonance imaging (DW-MRI). Moreover, a reasonable number of those patients had debris embolization on the filters of the CEPD. Pharmacological therapy with antithrombotic agents before, during, or after structural heart interventions is crucial and should be tailored to each patient’s risk of bleeding and ischemia. Close monitoring that includes a full neurological assessment and frequent follow-up visits with cardiac echocardiography are important.

**Summary:**

The risk of periprocedural stroke in left atrial and transcatheter mitral valve procedures is not negligible. Pharmacological therapy with antithrombotic agents before, during, or after structural heart interventions is important to mitigate the risk of stroke, especially the long-term risk. More prospective studies are needed to assess the efficacy of CEPD in such procedures.

## Introduction

Stroke remains one of the most serious and devastating complications of transcatheter heart interventions. Structural heart procedures are particularly hypothesized to be associated with ischemic stroke. Transcatheter aortic valve replacement (TAVR) is one of the most common cardiac procedures and is currently favored by the most recent American [1] and European [2] guidelines for the management of valvular heart disease for patients with severe aortic stenosis regardless of surgical risk. Despite the advancements in device technology and the improvement in operator expertise, it is still uncertain whether the risk of stroke after TAVR has decreased [3, 4].

Strokes associated with TAVR greatly affect patients’ quality of life and drive up healthcare expenses, with a sixfold increase in 30-day mortality rates [3, 5, 6]. Accurate diagnosis of strokes and silent cerebral lesions relies on comprehensive neurological evaluations and the type of imaging modality utilized. There is a pressing need to prevent stroke across various healthcare levels in numerous regions due to its profound impact on patients, their families, healthcare systems, and society in terms of both health and socioeconomic consequences [7, 8].

Cerebral embolic protection devices (CEPD) were initially developed to mitigate the risk of stroke associated with TAVR [9] by either filtering or deflecting potential cerebral emboli and were subsequently shown to be safe in various clinical settings. The efficacy of those devices during different cardiac interventions is yet to be fully demonstrated. Many studies have shed light on the use of CEPD during TAVR, however, the use of those devices during other structural heart interventions is not very well studied. Therefore, this review aims to discuss the current evidence on the use of CEPD during left atrial and mitral valve procedures.

### Stroke Risk

Debris embolization during structural heart procedures other than TAVR, such as transcatheter mitral valve therapies and left atrial appendage occlusion (LAAO), is known to occur [10–12]. Similar to TAVR, cerebral embolization during these procedures can either be asymptomatic and detectable only through brain imaging or can present as a major ischemic stroke. However, existing studies on cerebral embolization in non-TAVR procedures have often lacked formal neurological assessment. Consequently, the true incidence of neurological events may be underreported [9]. The risk of stroke is procedural; the highest risk of stroke is immediately post-procedure and then falls, and this is one of the reasons why the use of CEP in such procedures was suggested. Table 1 summarizes the stroke risk for LAAO, M-TEER) and TMVR.

**Table 1:** Stroke rates of mitral valve interventions and left atrial appendage occlusion

Procedure	Periprocedural stroke risk
Mitral Transcatheter Edge-to-Edge Repair	0.7-2.9%
Transcatheter Mitral Valve Replacement	~ 3%
Left Atrial Appendage Occlusion	0.2-1.2%

### Left Atrial Appendage Occlusion

Atrial fibrillation (AF) leads to a 4- to 5-fold higher risk of ischemic stroke and accounts for around 15% of ischemic strokes in the United States yearly [13–17]. For patients with nonvalvular AF and a moderate or high risk of stroke, it is recommended to use long-term anticoagulation to reduce the risk of stroke [18–21]. LAAO emerged as a treatment option for those patients who are poor candidates for long-term anticoagulation [22–25]. The left atrial appendage (LAA) is excluded from the systemic circulation, preventing thrombus formation and embolization, lowering the stroke risk [26–28].

The procedure itself, like other structural heart procedures, carries a risk of stroke. The incidence of stroke during LAAO ranges between 0.2% and 1.2% [9]. In a systematic review of LAAO procedures, using CEPD in 17 out of 58 patients, no periprocedural strokes were reported [29]. Evaluation with transcranial Doppler ultrasound (TCD) monitoring found microembolic signals in all patients during LAAO with the WATCHMAN device [30]. Moreover, new silent embolic lesions were found by diffusion-weighted magnetic resonance imaging (DW-MRI) in over one-third of patients within 24 h of the procedure [30]. The incidence of new brain lesions within 2 days following LAAO ranged between 4.8% and 52%[9 ,31, 32. In patients who are poor candidates for long-term anticoagulation, or in those for whom oral anticoagulation failed to dissolve the LAA thrombus, limited data is there on outcomes following LAAO when there is persistent thrombus within the LAA [9].

### Mitral Valve Interventions

Mitral valve procedures confer an inherent and non-negligible risk of ischemic stroke, albeit lower than those reported in TAVR. The COAPT trial reported a stroke rate of 0.7% at 30 days [33], and the MITRA-FR trial reported a periprocedural stroke rate of around 1.4% [34]. A systematic review and meta-analysis of 941 patients who underwent mitral transcatheter edge-to-edge repair (mTEER) found a periprocedural stroke incidence of 2.9% [35]. Other studies have compared different mitral valve interventions, including surgical and mTEER, and found stroke rates ranging between 0% and 10%[36]–[38]. Another systematic review and meta-analysis reported lower stroke rates with mTEER compared to surgical mitral valve repair/replacement [39].

In a small study that included 14 patients undergoing mTEER using CEPD (SENTINEL system), microscopic debris was detected in all filters, and that consisted mainly of acute thrombus or fragments of foreign material such as hydrophilic device coating [9, 11]. Similar to TAVR and LAAO, a study found that 87.5% of patients undergoing mTEER had cerebral ischemic injuries as assessed by pre- and post-procedural DW-MRI, in addition to a 16.6% incidence of overt stroke [40]. Furthermore, another study of 27 patients similarly found an incidence of 85.2% of new brain lesions in patients undergoing mTEER [41]. The incidence of stroke during other transcatheter mitral valve interventions, including percutaneous mitral annuloplasty and transcatheter mitral valve replacement (TMVR), is not very well studied. The incidence of acute stroke is reported to be around 3% [9].

Moreover, lacerating heavily calcified leaflets increases the risk of stroke [42, 43]. The LAMPOON procedure, which is often done in patients with extreme mitral valve calcification, can also increase the risk of stroke. Limited data suggests that the use of CEP for LAMPOON is warranted [44].

### Pharmacological Management

Device-based transcatheter interventions are supported by contemporary evidence for the management of patients with structural heart disease. As previously mentioned, these procedures carry an inherent risk of stroke, and thus antithrombotic therapy is required for patients undergoing such procedures before, during, or after the procedure to mitigate the risk of thromboembolic events [10], which inadvertently increases the risk of bleeding. However, pharmacotherapy is not as useful in mitigating the periprocedural stroke risk as it is in mitigating the long-term risk.

### Left Atrial Appendage Occlusion

Patients referred for LAAO represent a frail population of older patients with multiple comorbidities and a high risk of falls [45]. It is difficult to balance the ischemic and bleeding risk in this population. There is no consensus on periprocedural OAC during LAAO. OAC can either be continued throughout the procedure or interrupted depending on the preference of the operator [23, 46]. A few studies recommend pretreatment with aspirin at least one day before LAAO [23, 47], while others administered loading doses of aspirin and clopidogrel immediately following the procedure [48, 49]. Furthermore, unfractionated heparin is the preferred anticoagulant during the procedure, as it can be continuously monitored and can be rapidly reversed with protamine sulfate if needed [10]. The optimal timing to start the heparin infusion is debatable as some defer the infusion after septal puncture [50].

**Fig. 1:**
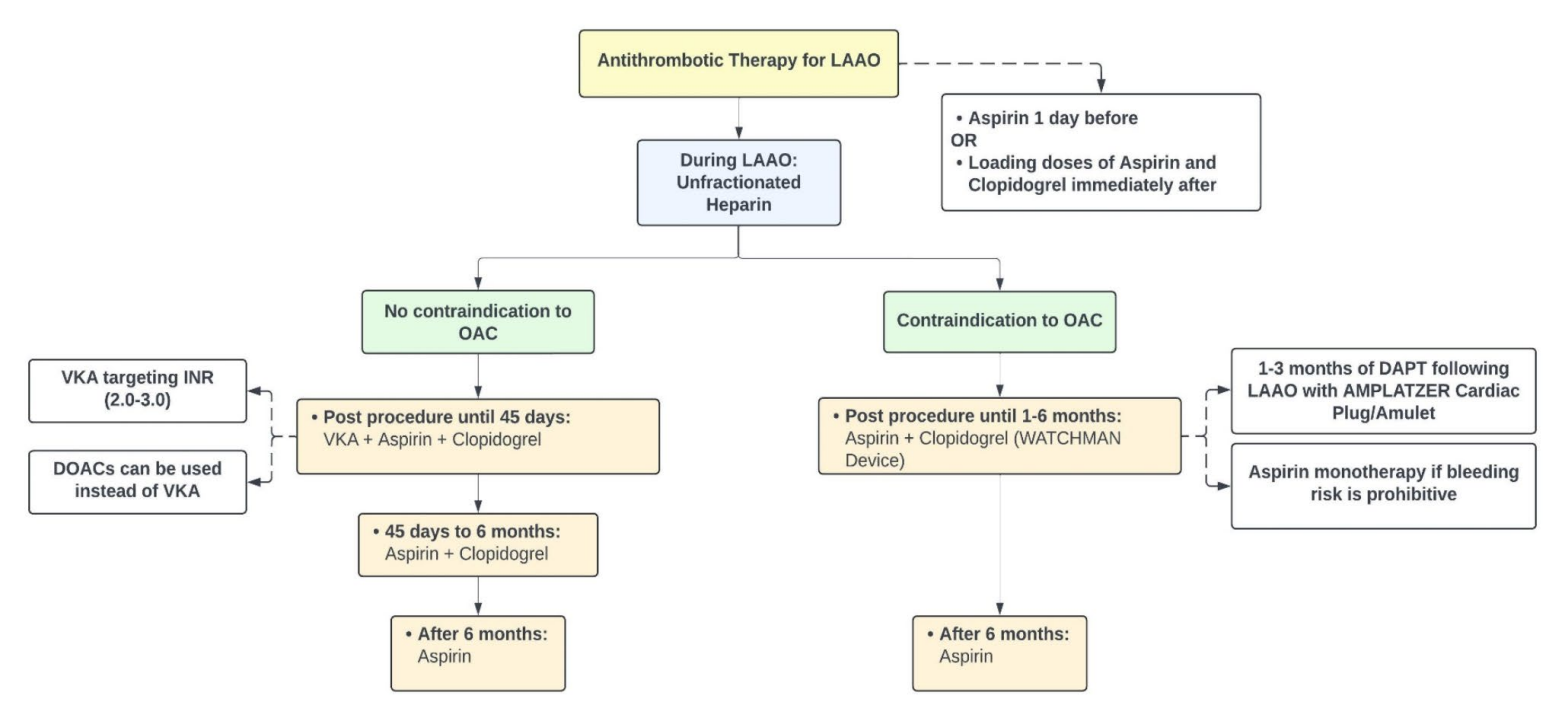
Antithrombotic therapy for left atrial appendage occlusion. *Abbreviations*: DAPT: dual antiplatelet therapy, DOAC: direct oral anticoagulants, INR: international normalized ratio, LAAO: left atrial appendage occlusion, OAC: oral anticoagulation, VKA: vitamin K antagonist

Antithrombotic therapy is necessary after the procedure to minimize the risk of thrombus formation on the left atrial side of the device, which could lead to embolization [50]. The highest risk of device-related thrombosis (DRT) is during the first 30–90 days after implantation and declines when device endothelialization is complete [51]. About 1 in 4 patients with DRT may develop a thromboembolic event [52]. There is no consensus on the optimal antithrombotic regimen following LAAO, but 3 main strategies exist. The first is a VKA-based strategy which consists of a short course of VKA (targeting INR 2.0–3.0) plus aspirin 81 mg/d for 45 days following LAAO with WATCHMAN device, followed by DAPT with aspirin 325 mg/d and clopidogrel 75 mg/d until 6 months after the procedure, followed by aspirin 325 mg/d, similar to the protocol of the PROTECT-AF trial [23]. An alternative strategy is using DOACs, but no trial powered for clinical outcomes was performed. The phase IIb randomized ADRIFT trial assessed rivaroxaban versus DAPT following LAAO and found that thrombin generation was lower in patients who received rivaroxaban [53]. A third strategy can be used in patients with a high bleeding risk or a contraindication to OAC. This strategy involves dual antiplatelet therapy with aspirin and clopidogrel for 1–6 months after LAAO with WATCHMAN (1–3 months after LAAO with AMPLATZER Cardiac Plug/Amulet), followed by long-term aspirin alone [48, 49, 54]. Alternatively, monotherapy with aspirin alone can be considered following LAAO if bleeding risk is deemed prohibitive [10]. If DRT occurs, therapeutic anticoagulation is advised to prevent embolization [52]. Figure 1 summarizes the antithrombotic management for patients undergoing LAAO.

### Mitral Valve Interventions

Current guidelines do not provide any recommendations for the antithrombotic management of mTEER [55, 56]. mTEER does not require anticoagulation therapy following the procedure since the device has low thrombogenicity. The most commonly used regimen consists of 1–6 months of dual antiplatelet therapy (DAPT) of aspirin and clopidogrel, followed by aspirin alone for 12 months or longer in patients without an indication for oral anticoagulants (OAC) [10]. This is derived from the protocol of the clinical studies assessing the device [33, 57, 58]. For patients with an indication for long-term OAC, vitamin K antagonists (VKA) are generally used, and direct oral anticoagulants (DOAC) can be used in select cases [10].

**Fig. 2:**
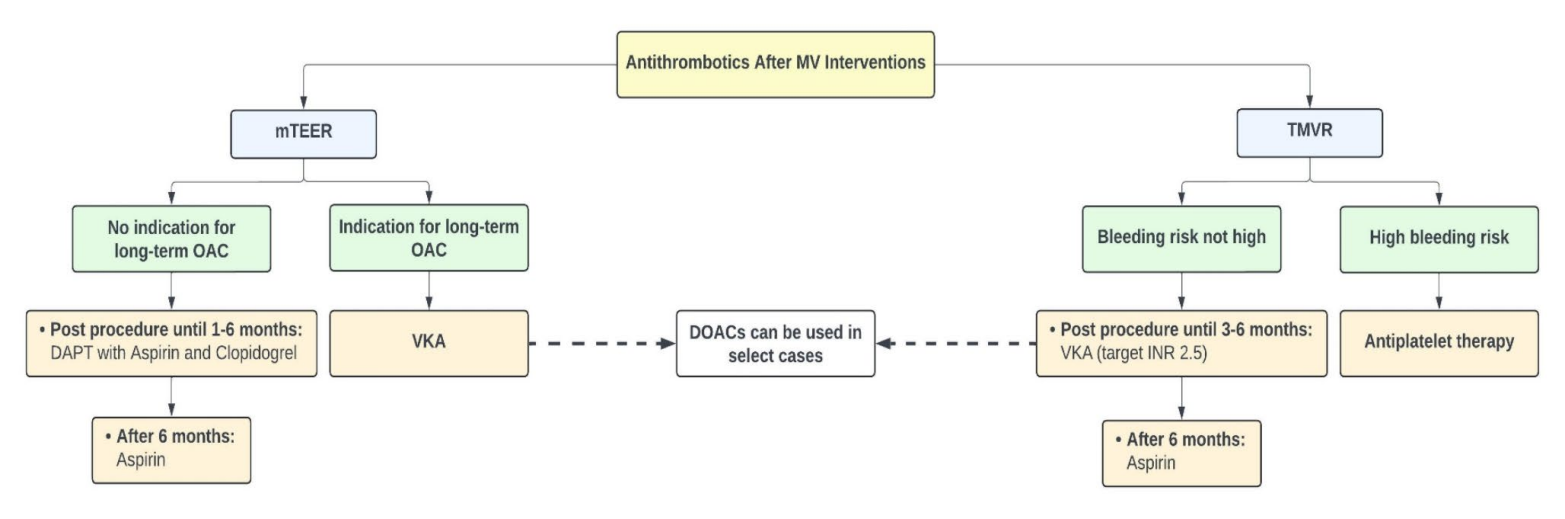
Antithrombotic therapy after mitral valve interventions. *Abbreviations*: DAPT: dual antiplatelet therapy, DOAC: direct oral anticoagulants, INR: international normalized ratio, mTEER: mitral transcatheter edge-to-edge repair, MV: mitral valve, OAC: oral anticoagulants, TMVR: transcatheter mitral valve replacement, VKAL vitamin K antagonist

No studies have prospectively evaluated antithrombotic strategies after TMVR. Therefore, considerable variation exists in practice. Available data showed a benefit from routine anticoagulation following TMVR, similar to the recommendations for surgical implantation of a bioprosthetic mitral valve [55, 56]. The guidelines recommend the use of OAC with VKA for 3–6 months with a target INR of 2.5 [55, 56]. Treatment duration is tailored to each patient’s bleeding and thromboembolic risks. Alternatively, DOACs can be used in place of VKA [10], although no studies have formally assessed their use. For patients who are at a high risk of bleeding, antiplatelet therapy can be used instead of OAC after TMVR; however, close follow-up is crucial [10]. Life-long low-dose aspirin is recommended for all patients with a bioprosthetic mitral valve [56]. It is still uncertain whether extending the use of OAC after 6 months is beneficial, and thus, close clinical and echocardiographic follow-up is needed to detect signs of bioprosthetic valve dysfunction. In the case of valve thrombosis, it is recommended to start or intensify anticoagulant therapy [55, 56]. Figure 2 summarizes the antithrombotic management of patients undergoing transcatheter mitral valve procedures.

## Conclusions

Structural heart procedures are associated with a risk of periprocedural ischemic stroke. Debris embolization occurs not only in TAVR, but also in other structural heart procedures like LAAO, mTEER, and TMVR. More prospective studies are needed to assess the efficacy of CEPD in LAAO, and mitral valve interventions, especially given that the risk of stroke is highest during the periprocedural period. Pharmacotherapy with antithrombotic agents is crucial before, during, or after such procedures, yet pharmacotherapy is still more useful in preventing late strokes than procedural strokes. Close monitoring and follow-up including a comprehensive neurological assessment are important.

## Data Availability

No datasets were generated or analysed during the current study.
